# Metformin attenuates O-GlcNAc modification to improve renal function *via* AMPK/mTOR signaling in diabetic nephropathy

**DOI:** 10.1016/j.jbc.2025.110909

**Published:** 2025-11-05

**Authors:** Bingxue Qi, Yang Chen, Yuejiao Lan, Yan Lou, Pingli Cui, Ruichen Yuan, Jingbo Zhao, Siyang Chai, Li Kang, Guannan Zhou, Shuangshuang Liu, Xiaodan Lu, Li-Hao Huang

**Affiliations:** 1 Precision Molecular Medicine Center, Jilin Province People's Hospital, Changchun, Jilin, China; 2 Clinical Medicine College, Changchun University of Chinese Medicine, Changchun, Jilin, China; 3 Department of Nephrology, The Second Hospital of Jilin University, Changchun, Jilin, China; 4 College of Integrated Traditional Chinese and Western Medicine, Changchun University of Chinese Medicine, Changchun, Jilin, China; 5 Division of Cellular and Systems Medicine, School of Medicine, University of Dundee, Dundee, UK; 6 Department of Gynecology, The Obstetrics and Gynecology Hospital of Fudan University, Shanghai, China; 7 Shanghai Key Laboratory of Metabolic Remodeling and Health, Institute of Metabolism and Integrative Biology, Liver Cancer Institute, Zhongshan Hospital, Fudan University, Shanghai, China

**Keywords:** diabetic nephropathy, mesangial cell hypertrophy, renal fibrosis, AMPK/mTOR, O-GlcNAc modification

## Abstract

Diabetic nephropathy is a growing global health challenge, significantly increasing the risks of hypertension and cardiovascular complications. Despite existing treatment options, none effectively promote renal repair or halt disease progression. We aimed to investigate the role of O-GlcNAc modification and the potential of Metformin as a therapeutic agent in diabetic nephropathy. In this study, we recruited diabetic nephropathy patients and treated them with Metformin. In addition, this research employed pharmacological and genetic methods to examine the impact of O-GlcNAc modification on diabetic nephropathy rat models and mesangial cells. Significant improvements in kidney function were observed in diabetic nephropathy patients treated with Metformin, as evidenced by reduced serum biomarkers and decreased mesangial matrix expression in renal biopsies. In the rat models, OSMI-1 treatment led to reduced renal fibrosis, inflammation, and pathological damage. Mechanistic investigations revealed that Metformin inhibits O-GlcNAc modifications, attenuates mesangial cell hypertrophy, and exerts its therapeutic effects through the AMP-activated protein kinase/mammalian target of rapamycin signaling pathway. These findings highlight Metformin as a promising therapeutic candidate for diabetic nephropathy. The study also sheds light on the novel role of O-GlcNAc modification in the pathogenesis of diabetic nephropathy, suggesting that targeting O-GlcNAc modifications could be a potential therapeutic strategy for diabetic nephropathy.

Diabetes mellitus (DM) is a growing global public health challenge. In 2021, the prevalence of DM was 5.29 billion worldwide, with projections indicating a rise to 1.31 billion by 2050 (1). Diabetic nephropathy (DN) has become the leading cause of chronic kidney disease (CKD) and represents one of the most prevalent microvascular complications associated with DM. It is primarily characterized by persistent proteinuria, hypertension, and progressive decline in renal function, potentially leading to end-stage renal disease (ESRD), which often necessitates dialysis or renal transplantation (2). Furthermore, DN significantly increases the risk of cardiovascular disease, along with its associated morbidity and mortality (3). Therefore, understanding the pathogenesis and exploring treatment methods for DN is crucial for early intervention, reducing complications and mortality, and alleviating the economic burden.

The pathogenesis of DN is characterized by a range of cellular processes, including increased inflammation (4), oxidative stress (5), decreased autophagy (6), dysregulated cell proliferation (7), mitochondrial dysfunction (8), and the accumulation of the extracellular matrix (ECM) and advanced glycation end products (AGEs) (9). These interconnected processes collectively activate profibrotic pathways, ultimately leading to renal fibrosis, dysfunction, and injury. In recent years, the role of O-GlcNAc modification in the development of DN has attracted growing attention (10). O-GlcNAc modification is a dynamic, reversible posttranslational modification where GlcNAc is attached to the hydroxyl groups of serine (Ser) and/or threonine (Thr) residues on target proteins. This process is regulated by two key enzymes: O-GlcNAc transferase (OGT) and O-GlcNAcase (OGA) (11). OGT facilitates the addition of GlcNAc from UDP-GlcNAc to specific protein residues, while OGA removes the modification (12). These enzymes play crucial roles in regulating protein interactions and are involved in essential biological processes (13). Advancing our understanding of the role of O-GlcNAc modification in DN is critical for developing future treatments to improve outcomes for affected patients. Our previous research has demonstrated that O-GlcNAc modifications of proteins contribute to renal cell damage, interstitial fibrosis, and the overall pathogenesis of DN (14). Moreover, current treatments for DN, including renin-angiotensin-aldosterone system inhibitors and sodium-glucose cotransporter 2 inhibitors, have shown promise in delaying disease progression, partly through the suppression of O-GlcNAc modifications (15, 16). Given these findings, directly targeting O-GlcNAc modifications could represent an effective therapeutic strategy for repairing and reversing renal injury.

Mesangial cells (MCs), derived from the posterior region of the renal interstitial tissue, are integral to the formation of the glomerular microvascular bed and the production of the mesangial matrix, which is crucial for maintaining glomerular homeostasis. In the early stages of DN, MCs undergo hypertrophy and secrete increased levels of matrix proteins, contributing to glomerular enlargement. Activation of the hexosamine biosynthetic pathway (HBP) in MCs by glucosamine induces a potent inhibition of proliferation, as evidenced by a significant reduction in cell number starting from 48 h of treatment, followed by growth arrest and hypertrophy (17). Notably, Fisi *et al.* reported that O-GlcNAc levels significantly increase during mitosis in HeLa cells compared to other cell cycle phases (18). These results implicate that O-GlcNAc modifications and HBP flux can be potential key regulators of cell cycle progression. Despite these findings, the mechanisms through which O-GlcNAc modifications contribute to MC hypertrophy remain poorly understood.

The AMP-activated protein kinase (AMPK)/mammalian target of rapamycin (mTOR) signaling pathway is a critical regulator of cellular metabolism and growth, and it plays a central role in the pathogenesis of DN. Studies indicate that Metformin exerts renoprotective effects by modulating the AMPK/mTOR axis, as evidenced by reduced podocyte apoptosis and proteinuria. Additionally, Metformin has been shown to decrease O-GlcNAc glycosylation levels, protecting against retinal cell death in diabetic mice (19). Whether Metformin can mitigate MC hypertrophy and renal fibrosis in DN by influencing the AMPK/mTOR pathway and modulating O-GlcNAc modifications warrants further exploration.

This study aimed to investigate the therapeutic effects of Metformin on MC hypertrophy and renal fibrosis in DN and its association with the O-GlcNAc modification. A clinical cohort of DN patients, with and without Metformin treatment, was assessed to evaluate posttreatment kidney function and injury biomarkers. Additionally, to elucidate the role of O-GlcNAc modification in DN pathogenesis, we utilized rat models of DN and *in vitro* cell culture systems. These complementary approaches provided mechanistic insights into the molecular pathways underlying Metformin's potential therapeutic benefits in DN.

## Results

### Metformin treatment improves DN and reduces O-GlcNAcylation in human kidney biopsies

**Figure 1:**
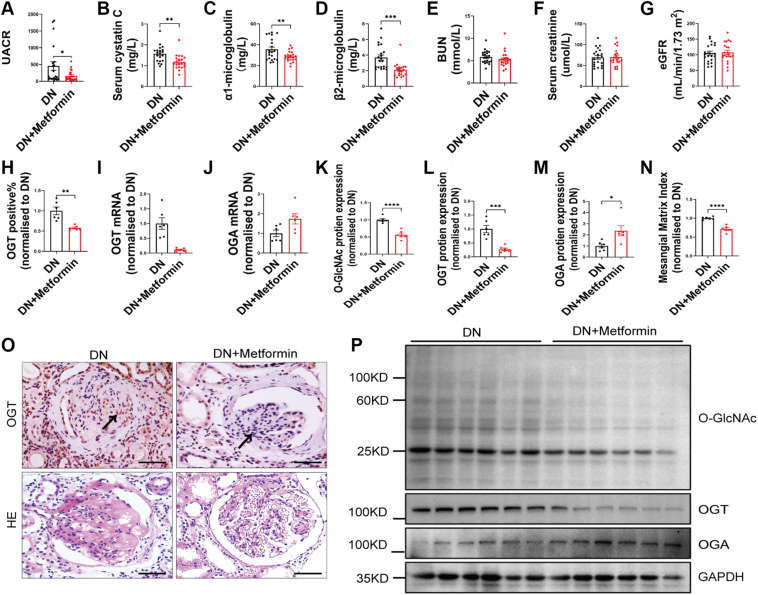
**Metformin treatment improves diabetic nephropathy and reduces O-GlcNAcylation in human kidney biopsies.***A–G*, the levels of UACR (*A*), serum cystatin C (*B*), α1-microglobulin (*C*), β2-microglobulin (*D*), BUN (*E*), serum creatinine (*F*), and eGFR (*G*) of DN patients with or without Metformin treatment, n = 20. *H*, quantification of OGT positive%, n = 6. *I–J*, quantification of OGT (I) and OGA (J) mRNA expression, n = 3 to 4. *K–M*, quantification of proteins O-GlcNAc (*K*), OGT (*L*), and OGA (M) n = 6. *N*, quantification of mesangial matrix index, n = 6. *O*, representative images of renal IHC staining for OGT and H&E staining of DN patients with or without Metformin treatment (the scale bar represents 100 μm). *P*, representative Western blot images of proteins O-GlcNAc, OGT, and OGA in human renal tissue, n = 6. Data are presented as mean ± SD. Significance analysis was performed with the unpaired Student's *t* test. ∗*p* < 0.05, ∗∗*p* < 0.01, ∗∗∗*p* < 0.001, and ∗∗∗∗*p* < 0.0001. DN, diabetic nephropathy; BUN, blood urea nitrogen; eGFR, estimated glomerular filtration rate; IHC, immunohistochemistry; OGA, O-GlcNAcase; OGT, O-GlcNAc transferase; UACR, urinary albumin-to-creatinine ratio.

To investigate whether Metformin improves kidney function and mitigates injury in DN, we recruited a cohort of patients with and without Metformin treatment and assessed posttreatment renal function and injury markers. In patients with early DN (urine albumin-to-creatinine ratio [UACR] >30 mg/g and estimated glomerular filtration rate (eGFR) >90 ml/min/1.73 m^2^), Metformin treatment reduced body mass index (BMI) and homeostatic model assessment for insulin resistance (HOMA-IR) (Table 1). Metformin significantly decreased UACR, serum cystatin C, α1-microglobulin, and β2-microglobulin levels compared to DN controls without Metformin treatment (Fig. 1, *A*–*D*). However, no significant changes were observed in blood urea nitrogen, serum creatinine, or eGFR markers of renal function (Fig. 1, *E–G*). In patients with biopsy-proven moderate DN (classes II and III), Metformin treatment reduced OGT expression, a key enzyme in O-GlcNAc modification, as confirmed by both immunohistochemistry (IHC) and quantitative real-time PCR analyses (Fig. 1, *H* and *I*). In addition, we also measured OGA levels, the enzyme responsible for removing O-GlcNAc modifications, and found that OGA mRNA expression was increased in these samples (Fig. 1*J*). This increase in OGA expression and the decrease in OGT levels suggest a shift in the balance of O-GlcNAc modification toward demodification. When we assessed the overall levels of O-GlcNAcylation, OGT, and OGA in human DN samples using Western blot, our results showed a significant reduction in total O-GlcNAcylation and OGT levels, an increase in OGA levels, as well as decreased MC proliferation and mesangial matrix accumulation, indicating that Metformin treatment not only affects the expression of OGT and OGA but also has a substantial impact on the overall O-GlcNAc modification status in DN samples (Fig. 1, *K*–*P*). Binary logistic regression analysis, with Metformin treatment as the dependent variable and BMI, HOMA-IR, and early kidney injury markers (UACR, serum cystatin C, α1-microglobulin, and β2-microglobulin) as independent variables, identified Metformin as a statistically significant independent factor associated with decreased UACR (Table 2). These findings suggest that Metformin mitigates kidney injury and improves renal pathology, which is associated with decreased OGT, increased OGA expression, and overall decreased O-GlcNAc modification levels in patients with early or moderate DN.

**Table 1:** Comparison of clinical parameters between two groups

Variables	DN	DN+Metformin	*P* Value
n (man/woman)	20 (8/12)	20 (7/13)	-
Age (years)	57.95 ± 1.83	53.70 ± 2.35	0.1613
Clinical course (years)	7.94 ± 1.53	8.38 ± 1.40	0.8363
SBP (mmHg)	132.7 ± 4.70	135.9 ± 3.98	0.6117
DBP (mmHg)	84.35 ± 2.40	84.15 ± 2.15	0.9508
BMI (kg/m^2^)	26.46 ± 0.59	24.60 ± 0.54	**0.0258**
TG (mmol/L)	2.70 ± 0.35	3.16 ± 0.63	0.5264
TC (mmol/L)	5.08 ± 0.21	5.11 ± 0.25	0.9250
LDL-c (mmol/L)	2.40 ± 0.14	2.48 ± 0.17	0.7153
HDL-c (mmol/L)	1.29 ± 0.09	1.32 ± 0.08	0.8302
HbA1C (%)	9.18 ± 0.41	8.81 ± 0.46	0.5586
FBG (mmol/L)	10.41 ± 0.90	10.28 ± 0.61	0.9075
PBG (mmol/L)	17.13 ± 1.27	14.32 ± 0.85	0.0735
HOMA-IR	7.69 ± 1.55	3.91 ± 0.46	**0.0270**

The values are expressed as mean ± SD.

Data in bold indicate statistically significant differences (*P* < 0.05).

SBP, systolic blood pressure; DBP, diastolic blood pressure; BMI, body mass index; TG, triglyceride; TC, total cholesterol; LDL-c, low-density lipoprotein-c; HDL-c, high-density lipoprotein-c; FBG, fasting blood glucose; PBG, postprandial blood glucose; HOMA-IR, homeostatic model assessment of insulin resistance.

**Table 2:** Binary regression analysis for Metformin treatment in patients with diabetic nephropathy

Variables	*B*	*SE*	*Waldχ 2*	*P*	*OR*
BMI	−0.009	0.442	0.000	0.983	0.991
HOMA-IR	−0.169	0.193	0.765	0.382	0.845
UACR	−0.036	0.017	4.323	**0.038**	0.965
Serum cystatin C	−1.172	2.216	0.280	0.597	0.310
α1-Microglobulin	−0.180	0.112	2.597	0.107	0.835
β2-Microglobulin	−0.501	0.447	1.258	0.262	0.606

The values are expressed as mean ± SD.

Data in bold indicate statistically significant differences (*P* < 0.05).

BMI, body mass index; HOMA-IR, homeostatic model assessment of insulin resistance; UACR, urinary albumin-to-creatinine ratio.

### Effects of O-GlcNAcylation on renal fibrosis, inflammation, and tubular damage, in a high-fat diet/streptozotocin-induced DN rat model

**Figure 2:**
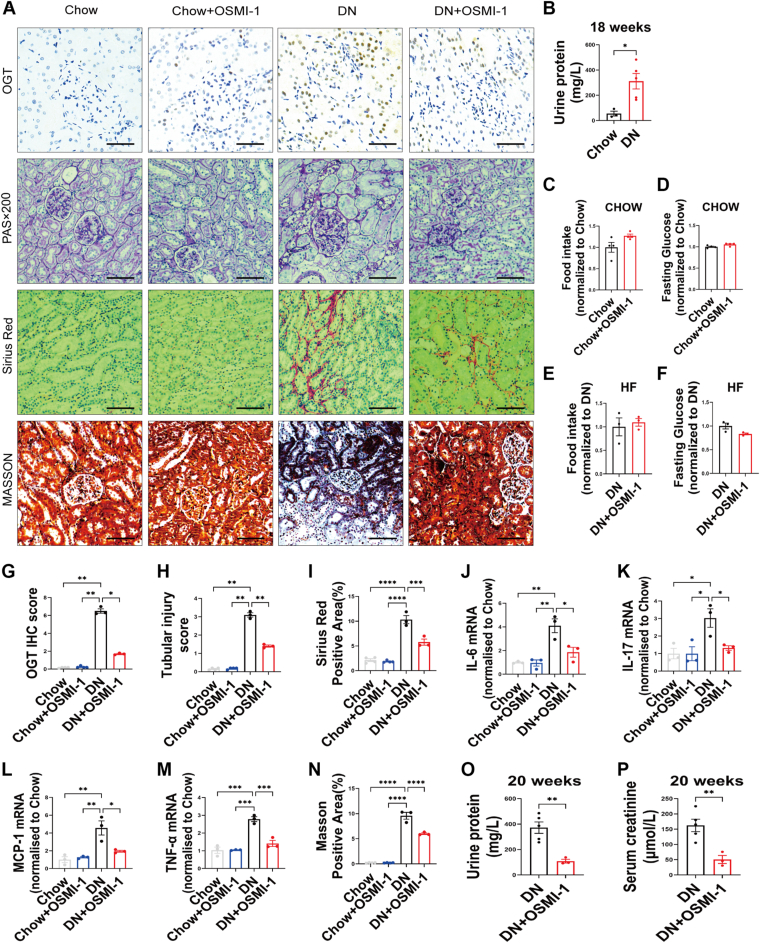
**Effects of O-GlcNAcylation on renal fibrosis, inflammation, and tubular damage in a high-fat diet/streptozotocin-induced kidney dysfunction rat model.***A*, representative images of renal IHC OGT, PAS, Sirius *Red*, and Masson staining in renal tissue of four groups (the scale bar represents 100 μm). *B*, urine protein concentration of normal control and DN rat model at 18 weeks, n = 3 to 5. *C–D*, food intake (*C*) and fasting glucose (*D*) of normal chow diet-fed SD rats with or without OSMI-1 injections, n = 3 to 4. *E–F*, food intake (*E*) and fasting glucose (*F*) of HF/STZ-induced DN rats receiving vehicle or OSMI-1 injections, n = 3. *G*, quantification of OGT IHC score, n = 3 to 4. *H*, tubular injury score, n = 3 to 4. *I*, quantification of collagen deposition, n = 3 to 4. *J–M*, mRNA expression of IL-6 (*J*), IL-17 (*K*), MCP-1 (*L*), andTNF-α (*M*) assessed by RT-PCR, n = 3 to 4. *N*, Masson-positive area was measured using the ImageJ program, n = 3 to 4. *O–P* urine protein concentration (*O*) and serum creatinine concentration (*P*) of HF/STZ-induced DN rats after receiving vehicle or OSMI-1 injections per day for 14 days. Data are presented as mean ± SD. Significance analysis was performed with one-way ANOVA (multiple comparisons). ∗*p* < 0.05; ∗∗*p* < 0.01; ∗∗∗*p* < 0.001; and ∗∗∗∗*p* < 0.0001. DN, diabetic nephropathy; HF, high fat; IHC, immunohistochemistry; IL-17, interleukin 17; IL-6, interleukin 6; MCP-1, monocyte chemoattractant protein-1; OSMI-1, O-GlcNAc transferase inhibitor-1; OGT, O-GlcNAc transferase; PAS, Periodic Acid Schiff; TNF-α, tumor necrosis factor-α.

Since we observed a correlation between improved renal pathology and reduced OGT protein expression in DN patients after Metformin treatment, we hypothesized that OGT plays a causal role in impairing kidney function. To test this hypothesis, we utilized a high-fat diet/streptozotocin (HFD/STZ)-induced DN model in Sprague-Dawley rats, which mimics the characteristics of human DN. By week 4, early renal lesions such as MC proliferation, mesangial matrix expansion, and basement membrane thickening could begin to manifest (20). Our results showed that by week 12, urine protein concentrations had exceeded 30 mg/L, confirming the successful establishment of the DN model (Fig. 2*B*). With this model, we examined renal function, pathological changes, inflammation, and fibrosis after administering the OGT inhibitor OSMI-1 at a dose of 1 mg/kg for 14 days. OSMI-1 treatment did not alter food intake or fasting glucose levels in either chow-fed or HFD-fed rats (Fig. 2, *C*–*F*). However, IHC analysis revealed that OSMI-1 significantly reduced OGT protein expression (Fig. 2, *A* and *G*). Further investigation into tubular damage, including epithelial cell vacuolar deformation/hypertrophy, tubular dilation, loss of brush border, and cell lysis, showed that OSMI-1 treatment alleviated these pathological changes in the HFD/STZ-induced DN model (Fig. 2, *A* and *H*). Additionally, OSMI-1 reduced collagen deposition and inflammation in the kidney cortex (Fig. 2, *A* and *I*–*M*), consistent with decreased renal fibrosis (Fig. 2, *A* and *N*). Markers of renal function, such as urine protein and serum creatinine concentrations, were also significantly improved (Fig. 2, *O* and *P*). In parallel with improved kidney pathology and function, OSMI-1 treatment enhanced glucose tolerance and insulin sensitivity (Fig. S1, *F*, *G*, *I* and *J*) but not reduced body weight (Fig. S1, *C* and *H*). OSMI-1 treatment does not cause significant damage to the liver or kidneys, as evaluated by tissue lactate dehydrogenase activity (Fig. S1, *K* and *L*). These findings demonstrate that inhibiting OGT in the kidney can potentially improve DN pathology, enhance renal function, and ameliorate glucose intolerance, insulin resistance.

### Knocking down *OGT* partially rescues MC volume and viability under high-glucose conditions

**Figure 3:**
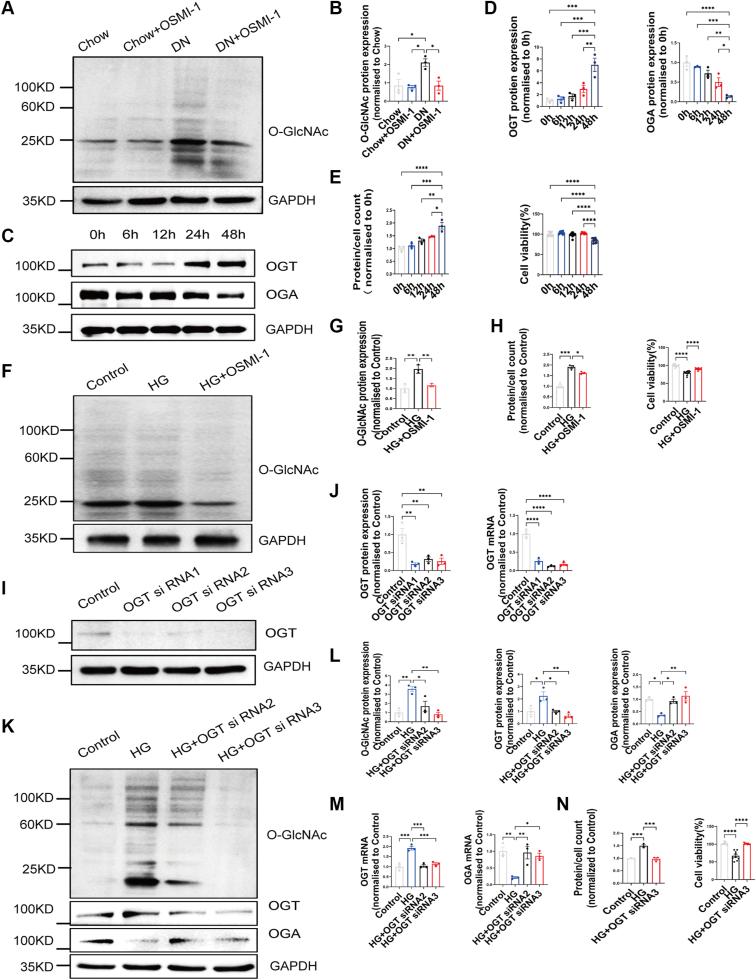
**Knocking down *OGT*partially rescues mesangial cell volume andviability under high-glucose conditions.***A*–*B*, representative Western blot images and quantification of total O-GlcNAcylation, n = 3. *C*–*D*, representative Western blot images and quantification of OGT and OGA protein expression in MCs of five groups according to hyperglycemic stimulation time, n = 3. *E*, the effect of glucose treatment on MCs volume and viability at different hyperglycemic stimulation times, n = 3 to 10. *F*-*G*, representative Western blot images and quantification of total O-GlcNAcylation in MCs of three groups according to whether or not OSMI-1 was added, n = 3. *H*, the effect of OSMI-1 treatment on MCs volume and viability under high-glucose conditions, n = 3 to 10. *I–J*, representative Western blot images and quantification of OGT protein and mRNA expression in MCs of four groups according to whether or not *OGT* gene silencing was performed. *K–L*, representative quantification and Western blot images of total O-GlcNAcylation, protein expression of OGT and OGA in renal tissue of four groups, n = 3. *M*, mRNA expression of OGT and OGA assessed by RT-PCR, n = 3. *N*, the effect of *OGT* gene silencing on MCs volume and viability under high-glucose conditions, n = 3 to 9. Data are presented as mean ± SD. Significance analysis was performed with one-way ANOVA (multiple comparisons). ∗*p* < 0.05; ∗∗*p* < 0.01; ∗∗∗*p* < 0.001; and ∗∗∗∗*p* < 0.0001. DN, diabetic nephropathy; MC, mesangial cell; OGA, O-GlcNAcase; OGT, O-GlcNAc transferase; OSMI-1, O-GlcNAc transferase inhibitor-1.

To further confirm the relationship between OGT inhibition and improved renal function, Western blot analysis was conducted on kidney tissues from HFD/STZ-induced DN rats treated with OSMI-1. The results revealed elevated total O-GlcNAcylation in the kidney of DN rats, which was effectively reversed following OSMI-1 treatment (Fig. 3, *A* and *B*). These findings suggest that OSMI-1 mitigates renal pathology, inflammation, dysfunction, and fibrosis through modulation of O-GlcNAc modification.

To mimic *in vivo* conditions, we established an *in vitro* MC culture system, exposing cells to different time periods with and without OSMI-1 treatment. Western blot analysis revealed that 48 h of high-glucose exposure resulted in increased OGT and decreased OGA protein expression in MCs (Fig. 3*D*). Additionally, high-glucose treatment increased MC volume (total protein-to-cell number ratio) and decreased MC viability, as assessed by the Cell Counting Kit-8 (CCK-8) assay (Fig. 3*E*). OSMI-1 treatment reversed these effects, decreasing total O-GlcNAcylation (Fig. 3, *F* and *G*), and partially rescuing MC volume and viability under high-glucose conditions (Fig. 3*H*). These *in vitro* results were consistent with the *in vivo* findings (Figs. 2 and 3*A*). To directly test OGT's role in MC function, we knocked down *OGT* using siRNA, reducing its mRNA expression to levels comparable to normal MCs under normal culture conditions (Fig. 3, *I* and *J*). Similar to OSMI-1 treatment, *OGT* knockdown decreased total O-GlcNAcylation and OGT protein and mRNA expression, while increasing OGA protein and mRNA expression. This was accompanied by decreased MC volume and increased MC viability after 48 h of high-glucose exposure (Fig. 3, *K*–*N*). These findings suggest that targeting *OGT* can improve damaged MCs, with the *in vitro* culture system effectively recapitulating the kidney injury observed in the rat DN model.

### Interaction between Akt/mTOR and O-GlcNAcylation influences renal injury, MC volume, and viability under high-glucose conditions

**Figure 4:**
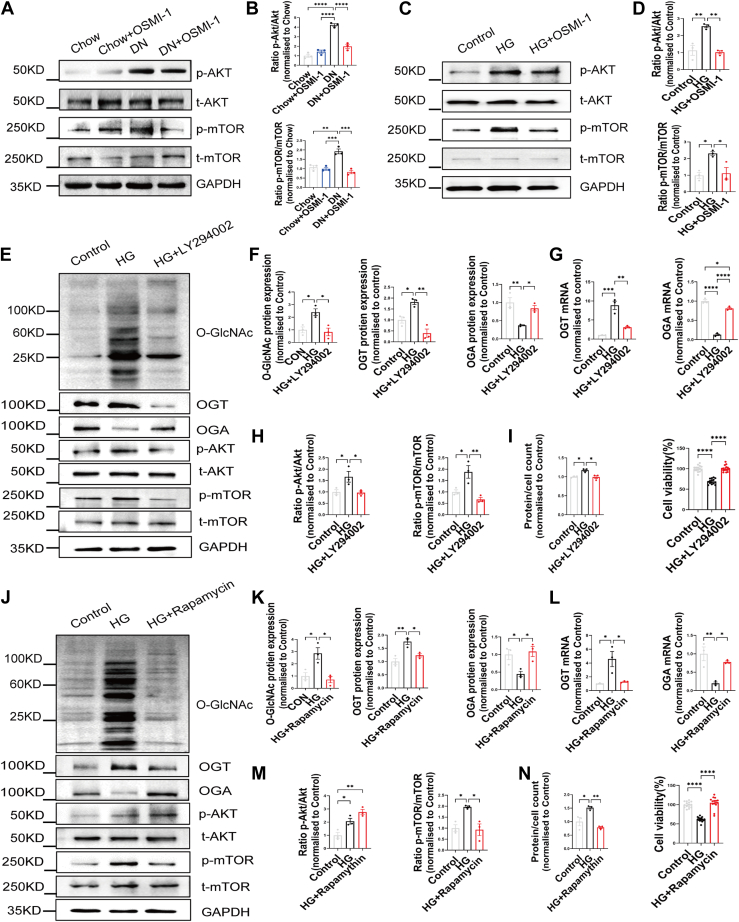
**Interaction between Akt/mTOR and O-GlcNAcylation influences renal injury, mesangial cell volume,andviability under high-glucose conditions.***A–B*, representative Western blot images (*A*) and quantification (*B*) of Akt/mTOR pathway proteins in renal tissue of four groups, n = 3. *C–D*, representative Western blot images (*C*) and (*D*) quantification of Akt/mTOR pathway proteins in MCs of three groups according to whether or not OSMI-1 was added, n = 3. *E*, Representative Western blot images of O-GlcNAc, OGT, OGA, and Akt/mTOR pathway proteins in MCs of three groups according to whether or not LY294002 was added, n = 3. *F*, representative quantification of O-GlcNAc, OGT, and OGA in MCs of three groups according to whether or not LY294002 was added, n = 3. *G*, mRNA expression of OGT and OGA assessed by RT-PCR, n = 3. *H*, quantification of Akt/mTOR pathway proteins in MCs of three groups according to whether or not LY294002 was added, n = 3. *I*, the effect of LY294002 treatment on MCs volume and viability under high-glucose conditions, n = 3 to 15. *J*, representative Western blot images of O-GlcNAc, OGT, and OGA and Akt/mTOR pathway proteins in MCs of three groups according to whether or not rapamycin was added, n = 3. *K*, representative quantification of O-GlcNAc, OGT, and OGA in MCs of three groups according to whether or not rapamycin was added, n = 3. *L*, mRNA expression of OGT and OGA assessed by RT-PCR, n = 3. *M*, quantification of Akt/mTOR pathway proteins in MCs of three groups according to whether or not rapamycin was added, n = 3. *N*, The effect of rapamycin treatment on MCs volume and viability under high-glucose conditions, n = 3 to 15. Data are presented as mean ± SD. Significance analysis was performed with one-way ANOVA (multiple comparisons). ∗*p* < 0.05; ∗∗*p* < 0.01; ∗∗∗*p* < 0.001; and ∗∗∗∗*p* < 0.0001. DN, diabetic nephropathy; MC, mesangial cell; mTOR, mammalian target of rapamycin; OGA, O-GlcNAcase; OGT, O-GlcNAc transferase; OSMI-1, O-GlcNAc transferase inhibitor-1.

The Akt/mTOR pathway has been implicated in the cellular response to high-glucose conditions (21). To investigate its activation in DN, we examined kidney tissues from HFD/STZ-induced DN rats and assessed whether OSMI-1 treatment could modulate this pathway *via* OGT inhibition. Western blot analysis revealed that high-glucose conditions significantly increased Akt and mTOR phosphorylation levels, which were reduced to baseline following OSMI-1 treatment (Fig. 4, *A* and *B*).

Consistent results were observed in an *in vitro* MC culture system. Under high-glucose conditions, OSMI-1 treatment reduced Akt and mTOR phosphorylation levels compared to untreated cells (Fig. 4, *C* and *D*). Furthermore, pharmacological inhibition of Akt with LY294002 or mTOR with rapamycin in high-glucose–treated MCs decreased total O-GlcNAcylation, OGT protein and mRNA expression, and increased OGA protein and mRNA expression (Fig. 4, *E*–*G*, *J*–*L*). It mitigated the high-glucose–induced increase in cell volume and decrease in cell viability (Fig. 4, *I* and *N*).

These findings suggest that the interaction between the Akt/mTOR pathway and OGT is a key driver of high-glucose–induced O-GlcNAc dysregulation, and that targeting this axis with pathway-specific inhibitors or OSMI-1 may restore MC function under diabetic conditions.

### AMPK/mTOR signaling pathway influences MC damage *via* O-GlcNAcylation under high-glucose treatment

**Figure 5:**
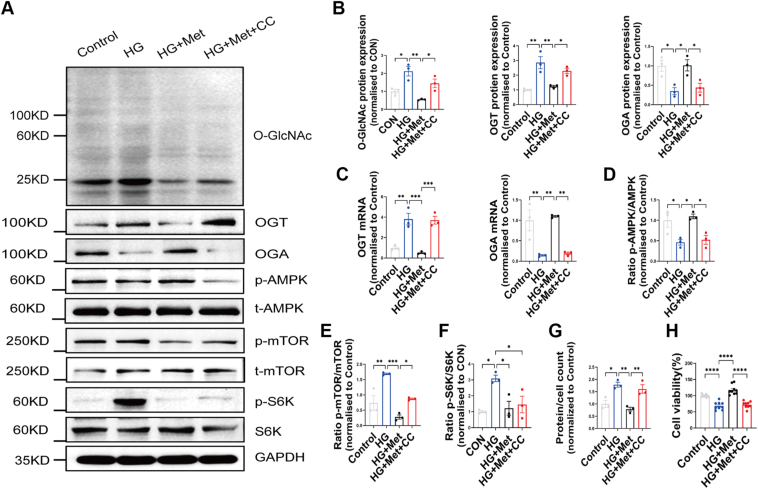
**AMPK/mTOR signaling pathway influences mesangial cell damage *via* O-GlcNAcylation under high-glucose treatment.***A*, representative Western blot images of four groups of O-GlcNAc, OGT, and OGA and AMPK/mTOR pathway proteins according to whether or not AMPK agonist and inhibitor were added, n = 3. *B*, representative quantification of O-GlcNAc, OGT, and OGA in MCs of four groups according to whether or not an AMPK agonist and inhibitor was added, n = 3. *C*, mRNA expression of OGT and OGA assessed by RT-PCR, n = 3. *D–F*, representative quantification of p-AMPK, p-mTOR, and p-S6K in MCs of four groups according to whether or not an AMPK agonist and inhibitor was added, n = 3. *G*, the effect of AMPK agonist and inhibitor treatment on MCs volume under high glucose conditions, n = 3. *H*, the effect of AMPK agonist and inhibitor treatment on MCs viability under high-glucose conditions, n = 3. Data are presented as mean ± SD. Significance analysis was performed with one-way ANOVA (multiple comparisons). ∗*p* < 0.05; ∗∗*p* < 0.01; ∗∗∗*p* < 0.001; and ∗∗∗∗*p* < 0.0001. AMPK, AMP-activated protein kinase; CC, compound C; MC, mesangial cell; Met, Metformin; mTOR, mammalian target of rapamycin; OGA, O-GlcNAcase; OGT, O-GlcNAc transferase.

Since we observed increased mTOR phosphorylation under high-glucose conditions, we investigated whether this was associated with reduced AMPK phosphorylation, a key metabolic regulator of cellular energy status, which could contribute to mTOR activation and subsequent protein synthesis. Western blot analysis revealed that high-glucose conditions decreased AMPK phosphorylation while increasing mTOR phosphorylation in MCs. These effects were reversed by Metformin treatment, an AMPK agonist (Fig. 5*A*, *D, E* and *F*).

Metformin treatment also partially alleviated the high-glucose–induced increase in MC volume and decrease in MC viability, potentially through the downregulation of total O-GlcNAcylation, OGT protein and mRNA expression, and upregulation of OGA protein and mRNA expression (Fig. 5, *A*–*C*). These findings are consistent with our clinical data and results from HFD/STZ-induced DN rats, where Metformin treatment was associated with decreased O-GlcNAcylation, reduced MC proliferation, and diminished mesangial matrix accumulation in DN patients (Fig. 1, *K*–*N*). To further validate the role of AMPK, we cotreated MCs under high-glucose conditions with Metformin and compound C, an AMPK inhibitor. Compound C reversed the reduction in mTOR phosphorylation achieved by Metformin, confirming that the effects of Metformin are mediated through AMPK activation (Fig. 5, *A*–*H*).

Together, these findings demonstrate that Metformin mitigates MC damage under high-glucose conditions by modulating the AMPK/mTOR pathway, leading to reduced O-GlcNAcylation and improved cell function.

## Discussion

Metformin, a first-line treatment for type 2 diabetes mellitus (T2DM,) exerts hypoglycemic effects by activating AMPK (22), inhibiting hepatic gluconeogenesis (23), and protecting β cells from oxidative stress and apoptosis (24). Studies have also shown that Metformin regulates cell cycle inhibitors, such as p21 and p27, through AMPK activation, thereby exerting cell cycle-blocking effects to inhibit cell proliferation (22, 25). Additionally, AMPK activation has been shown to inhibit O-GlcNAcylation in cells (26) and modulate the substrate selectivity of OGT (27). O-GlcNAc modification plays a pivotal role in the onset and progression of DN. In the kidney, elevated O-GlcNAc modification disrupts renal cell function and metabolic balance, leading to excessive ECM accumulation and thickening of the basement membrane. These pathological changes impair the renal filtration barrier, resulting in proteinuria and renal dysfunction (15). Whether Metformin improves kidney function by influencing O-GlcNAc modification or other pathways remains an area of active investigation.

The relationship between Metformin and kidney function is complex. While Metformin may pose risks, such as lactic acidosis in patients with moderate CKD and reduced eGFR, it has demonstrated renal protective effects in patients with an eGFR above 45 ml/min/1.73 m^2^ (28). These findings are consistent with current U.S. Food and Drug Administration guidelines recommending the use of Metformin in patients with an eGFR of ≥45 ml/min/1.73 m^2^, with caution below this threshold (29). In this study, we enrolled patients with early DN (UACR >30 mg/g, eGFR >90 ml/min/1.73 m^2^) and patients with moderate DN (classes II and III of Tervaert's classification, eGFR >45 ml/min/1.73 m^2^). Metformin treatment significantly reduced UACR, serum cystatin C, α1-microglobulin, and β2-microglobulin levels in early-stage DN patients. In moderate DN, Metformin treatment not only affects the expression of OGT and OGA but also has a substantial impact on the overall O-GlcNAc modification status in DN samples, along with decreased MC proliferation and mesangial matrix accumulation, suggesting that its renoprotective effects are mediated through O-GlcNAc modification. Consistent with these findings, OGT inhibition with OSMI-1 in HFD/STZ-induced DN rats alleviated renal damage, reduced ECM accumulation, and decreased renal inflammation and fibrosis. Interestingly, a recent study revealed that O-GlcNAc modification is elevated in diabetic retinopathy, contributing to retinal neurodegeneration and microangiopathy. Notably, these pathological changes were ameliorated following Metformin treatment (30). These findings provide valuable clinical insights into the role of O-GlcNAc modification in diabetic complications and highlight Metformin's potential as a therapeutic agent for managing diabetic retinopathy.

MCs, originating from the posterior renal interstitium, play a vital role in forming the glomerular microvasculature and synthesizing the mesangial matrix, which is essential for maintaining glomerular homeostasis. However, MC hypertrophy and increased secretion of matrix proteins result in glomerular enlargement, one of the earliest alterations observed in DN. In this study, we demonstrated that high-glucose conditions induce MC hypertrophy both *in vivo* and *in vitro*, a process closely associated with changes in O-GlcNAc modification. The AMPK/mTOR pathway, a critical regulator of cellular metabolism and growth, has significant implications in the progression of DN (31, 32). To investigate its role, we analyzed the modulation of AMPK and mTOR phosphorylation levels and their effects on O-GlcNAc modification in MCs under high-glucose conditions. Treatment with Metformin, an AMPK agonist, activated AMPK, inhibited mTOR signaling, downregulated OGT expression, and upregulated OGA expression. These changes partially reversed the increase in MC volume and decrease in MC viability caused by high-glucose conditions. Conversely, the use of compound C, an AMPK inhibitor, negated these protective effects, further confirming the pathway's role. Our findings indicate that the AMPK/mTOR signaling pathway is a key mechanism by which Metformin exerts protective effects in MCs *via* modulation of O-GlcNAc modification. These results provide valuable insights into the cellular mechanisms underlying Metformin's therapeutic effects on diabetic kidney injury and its potential to target O-GlcNAc modification in DN.

In this study, we found that inhibition of OGT with OSMI-1 in HFD/STZ-induced DN rats alleviated renal damage, reduced ECM accumulation, and decreased renal inflammation and fibrosis by downregulating the Akt/mTOR pathway. This finding is consistent with previous studies, showing that O-GlcNAcylation is associated with the Akt/mTOR pathway in conditions such as thrombosis, inflammation, and cancer (33). Furthermore, in our study, pharmacological inhibition of Akt with LY294002 or mTOR with rapamycin downregulated the Akt/mTOR pathway in high-glucose–treated MCs, leading to decreased O-GlcNAcylation. Similarly, in breast cancer cells, PI3K and mTOR inhibitors have been shown to reduce overall O-GlcNAcylation levels (34). These findings underscore a dynamic interplay between the AKT/mTOR pathway and protein O-GlcNAcylation, two pivotal regulators of cellular metabolism and stress adaptation (35). The marked decrease in total O-GlcNAc levels following rapamycin treatment indicates that mTOR signaling exerts substantial control over global O-GlcNAc modification. Given that rapamycin is a well-characterized mTOR inhibitor known to modulate protein synthesis, autophagy, and metabolic reprogramming, this observation suggests that mTOR activity is tightly linked to O-GlcNAc homeostasis (36, 37). Mechanistically, mTOR inhibition may influence O-GlcNAcylation through several routes. One possibility is that reduced mTOR activity alters the expression or catalytic function of OGT to target proteins (36, 37, 38). Alternatively, suppressing mTOR signaling could impact intracellular UDP-GlcNAc pools, the substrate for OGT, by reshaping nutrient flux or HBP activity (36). Disentangling these mechanisms will be important to fully define how nutrient-sensing and post-translational modification networks integrate at the cellular level. From a broader perspective, the sensitivity of O-GlcNAc modification to mTOR inhibition highlights a potential therapeutic intersection between these two signaling axes. Aberrant O-GlcNAcylation is implicated in diverse pathologies, including diabetes, cardiovascular diseases, neurodegenerative disorders, and cancer (35), all of which feature metabolic dysregulation. Thus, pharmacological modulation of mTOR activity by rapamycin or its derivatives may provide a strategy to normalize O-GlcNAc levels and restore metabolic balance. Further investigation into this crosstalk could reveal new opportunities for targeting mTOR–O-GlcNAc signaling in metabolic diseases.

Recent studies have highlighted the therapeutic potential of dapagliflozin, a sodium-glucose cotransporter 2 inhibitor, in DN. It has been shown to enhance autophagy and reduce apoptosis by modulating the AMPK/mTOR pathway (31). Additionally, Krit *et al.* reported that the combined use of dapagliflozin and Metformin exerts synergistic benefits in DN in rats, including reductions in oxidative stress, inflammation, and apoptosis, alongside enhanced autophagy through modulation of the AMPK/mTOR/SIRT1 axis (39). Future investigations into the effects of dapagliflozin on O-GlcNAc modification levels following AMPK activation could provide valuable insights. Specifically, exploring how dapagliflozin influences mTOR signaling and GlcNAc modification may uncover novel mechanisms and therapeutic targets for managing DN.

In summary, our findings demonstrate that Metformin treatment effectively decreased O-GlcNAcylation, suppressed MC proliferation, and attenuated mesangial matrix accumulation in kidney biopsies from patients with moderate DN. In HFD/STZ-induced DN rats, OGT inhibition with OSMI-1 significantly alleviated renal pathological damage, reduced ECM accumulation and inflammation, and prevented renal fibrosis and dysfunction. These results were further supported by *in vitro* studies in MCs, confirming that the protective effects under high-glucose conditions are mediated through OGT regulation *via* the AMPK/mTOR pathway. Collectively, our study identifies OGT as a promising therapeutic target for DN.

## Experimental procedures

### Patients

A total of 40 participants were recruited from the Department of Endocrinology at Jilin Province People's Hospital between February 2023 and February 2024. Inclusion criteria were individuals aged 18 to 75 years newly or previously diagnosed with T2DM according to the WHO's 1999 Diagnostic Criteria for T2DM (40). Exclusion criteria included acute kidney injury, pregnant or lactating women, patients with malignancies, cardiovascular or respiratory diseases, hematological disorders, immune system diseases, primary kidney diseases, hypertension, severe mental illness, or inability to cooperate with researchers. All participants received insulin for blood glucose control, statins for blood lipid management, and calcium antagonists for blood pressure regulation. Each participant provided informed consent in compliance with the Declaration of Helsinki, and the study was approved by the Research Ethics Committee of Jilin Province People's Hospital (2023153).

### Patient experiment design 1#

To examine Metformin's effect on renal injury in patients with initial DN, identified by a persistent UACR exceeding 30 mg/g and an eGFR of ≥90 ml/min/1.73 m^2^ (41), participants were categorized into two groups based on their basic treatments: the DN group without Metformin (n = 20) and the DN + Metformin group (500 mg orally twice daily) (n = 20).

### Measurement of clinical parameters

Clinical parameters collected included demographic information (sex, age, clinical course), physiological measurements (systolic blood pressure, diastolic blood pressure, BMI), lipid profiles (triglyceride, total cholesterol, low-density lipoprotein cholesterol, high-density lipoprotein cholesterol), and measurements of hemoglobin A1c, fasting blood glucose, postprandial blood glucose, fasting plasma insulin, serum creatinine, and blood urea nitrogen, α1-microglobulin, β2-microglobulin, UACR, and eGFR. The eGFR was calculated using the Chronic Kidney Disease Epidemiology Collaboration formula (42), and insulin resistance was determined using the HOMA-IR index (43).

### Patient experiment design 2#

To investigate Metformin's impact on renal pathological changes, focusing on O-GlcNAc modification in patients with moderate DN (confirmed by biopsy and classified as stages II and III with eGFR ≥45 ml/min/1.73 m^2^), participants were allocated into two groups: the DN group without Metformin (n = 6) and the DN + Metformin group (500 mg orally twice daily) (n = 6).

### Animals

Animal experiments complied with the Institutional Animal Care and Use Committee of Changchun University of Chinese Medicine and followed the NIH guidelines. Male rats were housed in an air-conditioned room with a 12-h light/dark cycle and free access to water and food. They were fed either a high-fat or a chow diet starting at 6 weeks of age for 14 weeks and studied at 20 weeks. Five-week-old WT male Sprague-Dawley rats were obtained from the Animal Experiment Center of Jilin University. Twelve rats were given an HFD after an initial week of adaptive feeding, and a single intraperitoneal injection of low-dose STZ was administered at 6 weeks of age to induce a type 2 diabetic rat model. The remaining rats served as the control group. After 12 weeks of treatment, urine was collected, and a urine protein concentration of more than 30 mg/l indicated a successful DN rat model. DN model rats received injections of either vehicle (10 mmol/l histidine, 130 mmol/l NaCl at pH 6.5) or OSMI-1 (TargetMol; #T16409), a cell permeable OGT inhibitor, at a dose of 1 mg/kg intraperitoneally per day for 2 weeks.

### Animal experiment design

The study aimed to investigate the role of O-GlcNAc modification in kidney injury associated with DN in rats. Rats were randomly assigned to four groups: Control rats fed a normal chow diet (chow), control rats fed a normal chow diet and receiving OSMI-1 injections (chow + OSMI-1), DN rats fed an HFD and receiving vehicle injections (DN + vehicle), and DN rats fed an HFD and receiving OSMI-1 injections (DN + OSMI-1).

### Body weight and food intake

Body weights were measured weekly, and the basal food intake of the rats was monitored continuously for 7 days to calculate the daily basal food intake.

### Glucose tolerance test and insulin tolerance test

For the glucose tolerance test, rats were fasted for 16 h before being injected intraperitoneally with D-glucose, and blood glucose levels were monitored at various time points. For the insulin tolerance test, mice were fasted for 6 h before being injected intraperitoneally with recombinant human insulin, and blood glucose levels were monitored.

### Urine protein concentration and renal function measurement

Urine protein concentration was assessed using a Urine Protein Colorimetric Assay Kit (#E-BC-K252-M, Elabscince). Similarly, serum creatinine levels were determined using a Creatinine Colorimetric Assay Kit (#E-BC-K188-M, Elabscince).

### Histology and IHC

Paraffin-embedded rat kidney sections, 5-μm thick, were prepared following a standard protocol. The sections were stained with Periodic acid-Schiff using the PAS Stain Kit (#ab150680; Abcam), Sirius Red staining using Direct Red 80 (#365548; Sigma) and Picric acid (#P6744; Sigma), and Masson's trichrome staining using the Masson Staining Kit (#C0189, Beyotime). Immunohistochemical staining for OGT was performed using anti-OGT (#R25212, Zen Bio, 1:100), horseradish peroxidase-linked anti-rabbit (#7074S, Cell signaling, 1:1000), and DAB Substrate kit (#D3939, Sigma) as previously described (44). The antibody was previously verified or stained with a positive control. Images were captured and analyzed in a blinded manner. Images were captured with a camera mounted on a NOVEL microscope (#NIB610). The degree of tubular damage was scored using the system described by Haut *et al.* (45). Quantification of Sirius red, Masson and OGT immunohistochemical staining was assessed in the cortex area using ImageJ Software (https://imagej.net/ij/).

### RNA extraction and real-time RT-PCR

Total RNA was extracted from rat kidneys using TRIzol reagent (#R401-01-AA, Vazyme) and reverse transcribed into complementary DNA with HiScript III RT SuperMix for quantitative real-time PCR (#R323-01, Vazyme). mRNA expression levels were assessed by real-time quantitative RT-PCR, normalized to 18S expression levels, and quantified using the 2^−ΔΔCT^ method. The sequences of the specific primers are detailed in Table S1.

### Cell culture

The mouse MC line was a generous gift from Dr Lining Miao. The authenticity of the MC line was confirmed by short tandem repeat profiling, and the results are consistent with the ICLAC database. Additionally, the MC line was regularly tested for *mycoplasma* contamination using a commercially available PCR-based detection kit, and no contamination was detected throughout the study. The cells were maintained in Dulbecco's modified Eagle's medium containing 10% fetal bovine serum at 37 °C under 5% CO_2_. MCs were cultured *in vitro* for 3 to 10 generations, and experimental setups were used to investigate the role of O-GlcNAc modification in hyperglycemia-induced MC hypertrophy and proliferation inhibition.

### Cell experiment design 1#

To investigate the optimal timing for the effects of hyperglycemia-induced MC hypertrophy and proliferation inhibition, as well as their relationship with O-GlcNAc modification, MCs were categorized into groups based on the duration of hyperglycemic stimulation: (1) 0-h group, (2) 6-h group, (3) 12-h group, (4) 24-h group, and (5) 48-h group.

### Cell experiment design 2#

To evaluate the impact of OGT inhibition on O-GlcNAc modification and MC damage under high-glucose treatment over 48 h, MCs were categorized into three groups based on glucose treatment and the addition of the OGT inhibitor OSMI-1: (1) Control group (5.5 mmol L^−1^ glucose); (2) HG group (30 mmol L^−1^ glucose); and (3) HG + OSMI-1 group (30 mmol L^−1^ glucose + 5 mmol L^−1^ OSMI-1).

### Cell experiment design 3#

To further confirm the effects of O-GlcNAc modification modulated by *OGT* gene on MC damage under high-glucose treatment over 48 h, MCs were divided into four groups: (1) Control group (5.5 mmol L^−1^ glucose + 50 nmol L^−1^ nontargeting siRNA); (2) HG group (30 mmol L^−1^ glucose); (3) HG+OGT siRNA2 group (30 mmol L^−1^ glucose + 50 nmol L^−1^ *OGT*-specific siRNA2, sequence: 5′- GUGCACUGUUCAUGGAUUA-3′); and (4) HG+OGT siRNA3 group (30 mmol L^−1^ glucose + 50 nmol L^−1^ *OGT*-specific siRNA3, sequence: 5′- CUACGAGCAAGGCCUAAUA-3′).

### Cell experiment design 4#

To observe the changes of Akt/mTOR pathway in MCs under high-glucose condition over 48 h post-Akt inhibitor treatment, MCs were divided into three groups: (1) Control group (5.5 mmol L^−1^ glucose); (2) HG group (30 mmol L^−1^ glucose); and (3) HG+LY294002 group (30 mmol L^−1^ glucose + 30 μmol L^−1^ LY294002 (AKT inhibitor)).

### Cell experiment design 5#

To observe the changes of Akt/mTOR pathway in MCs under high-glucose condition over 48 h post-mTOR inhibitor treatment, MCs were divided into three groups: (1): Control group (5.5 mmol L^−1^ glucose); (2) HG group (30 mmol L^−1^ glucose); and (3) HG+rapamycin group (30 mmol L^−1^ glucose + 500 nmol L^−1^ rapamycin (mTOR inhibitor)).

### Cell experiment design 6#

To further observe the changes of AMPK/mTOR pathway in MCs under high-glucose condition over 48 h post-AMPK agonist and inhibitor treatment, MCs were divided into four groups: (1) Control group (5.5 mmol L^−1^ glucose); (2) HG group (30 mmol L^−1^ glucose); (3) HG+Metformin group (30 mmol L^−1^ glucose + 2.5 mmol L^−1^ Metformin); and HG+Metformin+CC group (30 mmol L^−1^ glucose + 2.5 mmol L^−1^ Metformin + 10 μmol L^−1^ compound C).

### Assessment of cell hypertrophy and viability

MCs were treated according to various experimental setups and then lysed using 1X radio-immunoprecipitation assay lysis buffer (#BL504A; Biosharp), supplemented with 1% PMSF and a protease and phosphatase inhibitor cocktail (#PR20038; Proteintech). Protein content was quantified using the BCA assay kit (catalog number #PK10026; Proteintech), and cell hypertrophy was assessed by determining the ratio of total protein to cell number (46). The CCK-8 (#C6005; NCM Biotech) assay was utilized to assess cell viability. A density of 2 × 10ˆ3 cells per well was seeded into each well of 96-well plates according to various experimental setups with different treatments. Following treatment, 10 μl of the CCK-8 reagent was added to each well, and the plates were incubated at 37 °C for 1 to 4 h in the dark. The absorbance of each well was measured using a microplate reader at a wavelength of 450 nm (Varioskan Flash, Thermo Fisher Scientific).

### Western blotting

Kidney tissue and cell homogenates were prepared using 1X radio-immunoprecipitation assay Lysis Buffer (#BL504A; Biosharp), supplemented with 1% PMSF and a protease and phosphatase inhibitor cocktail (#PR20038; Proteintech). After centrifugation at 13,000 rpm for 15 min, the supernatant was collected. Protein concentrations were determined using a BCA assay kit (#PK10026; Proteintech). Protein samples (30 μg) were loaded onto a 10% SDS-PAGE gel, run at 150 V, and then transferred onto nitrocellulose membranes (#106000023, Cytiva AmershamTM). The following primary antibodies were used to detect the respective proteins after incubation overnight at 4 °C in 5% BSA: O-GlcNAc (#65292-1-lg, proteintech, 1:1000), OGT (#R25212, Zen Bio, 1:1000), OGA (#R24971, Zen Bio, 1:1000), Phospho-AKT (Ser473) (#310021, Zen Bio, 1:1000), AKT (#R23412, Zen Bio, 1:1000), Phospho-mTOR (Ser2448) (#381557, Zen Bio, 1:1000), mTOR (#380411, Zen Bio, 1:1000), Phospho-AMPK alpha 1/2 (Thr183/Tyr172) (#340763, ZenBio, 1:1000), AMPK alpha (#23314, ZenBio, 1:1000), Phospho-P70 S6K (Thr389) (#AF5899, Beyotime, 1:1000), P70 S6K (#AF0258, Beyotime, 1:1000), and GAPDH (#TA802519, Origene, 1:1000). Membranes were washed six times with tris-buffered saline containing 0.1% Tween-20 containing 0.1% Tween-20 and incubated with horseradish peroxidase-goat-anti-rabbit secondary antibodies (#E-AB-1003, Elabscience) for 1 h at room temperature. All the antibodies were previously verified or ran with a positive control. Densitometric quantification was performed on scanned images using ImageJ software.

### Statistical analysis

Data were expressed as mean ± SD and analyzed using Prism GraphPad Software version 10 (https://www.graphpad.com/scientific-software/prism/). All the individual data points are independent biological replicates. The unpaired, two-tailed Student's *t* test was used for two-group comparisons, and one-way ANOVA followed by Tukey's multiple comparisons test was used for multiple-group comparisons. Data were considered statistically significant at *p* < 0.05.

## Data availability

All data will be made available on request.

## Supporting information

This article contains supporting information.

## Conflict of interest

The authors declare that they have no conflicts of interest with the contents of this article.
