# Supplementary material for: Metformin attenuates O-GlcNAc modification to improve renal function via AMPK/mTOR signaling in diabetic nephropathy
Source: J Biol Chem. 2025 Nov 5;301(12):110909. doi: 10.1016/j.jbc.2025.110909 (PMC12719668; doi:10.1016/j.jbc.2025.110909)
Supplement: Supporting Figure and Table [file mmc1.docx]

Supplementary materials


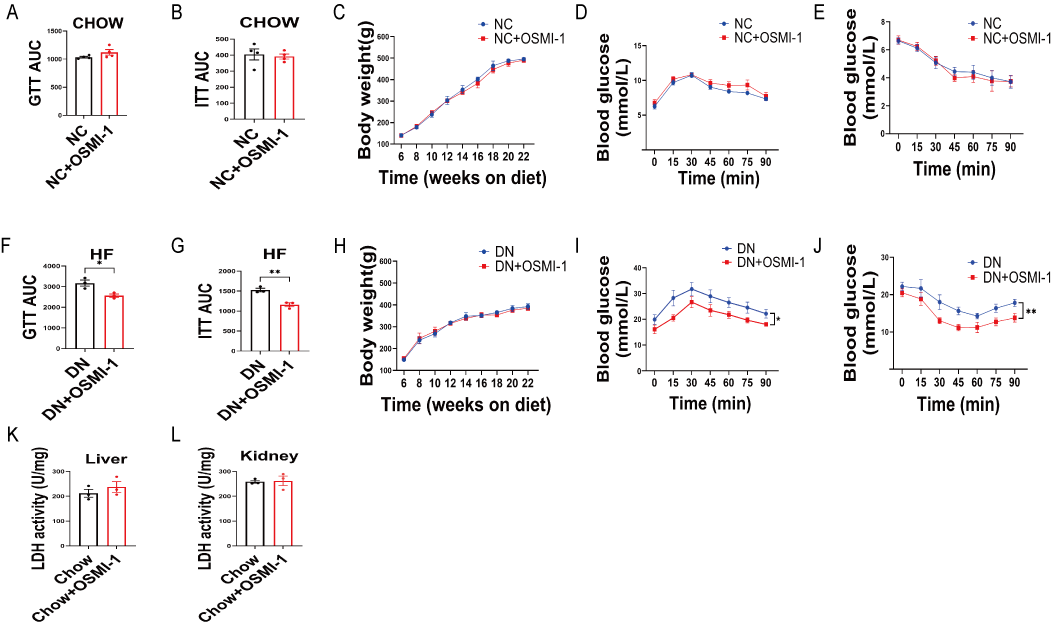


**Fig. S1.** OGT Inhibition alleviate impaired glucose tolerance and insulin resistance in HF/STZ-induced DN rats. *A-B*, GTT AUC (*A*) and ITT AUC (*B*) of normal chow diet-fed SD rats with or without OSMI-1 injections, n=4. *C-E*, Body weight (*C*), GTT (*D*) and ITT (*E*) of normal chow diet-fed SD rats with or without OSMI-1 injections, n=4. *F-G*, GTT AUC (*F*) and ITT AUC (*G*) of HF/STZ-induced DN rats receiving vehicle or OSMI-1 injections, n=3. (*H-J*) Body weight (*H*), GTT (*I*) and ITT (*J*) of HF/STZ-induced DN rats receiving vehicle or OSMI-1 injections, n=3. *K-L*, LDH activity in the liver (*K*) and kidneys (*L*) of normal chow diet-fed SD rats with OSMI-1 injections, n=3. Data are presented as mean ± SD. Significance analysis was performed with the unpaired Student's *t* test. **p* < 0.05; ***p* < 0.01.

| rats *IL-6* | Forward | CCGGAGAGGAGACTTCACAG |
| --- | --- | --- |
|  | Reverse | GCCATTGCACAACTCTTTTCTC |
| rats *IL-17* | Forward | TTTAACTCCCTTGGCGCAAAA |
|  | Reverse | CTTTCCCTCCGCATTGACAC |
| rats *TNF-α* | Forward | ACTGAACTTCGGGGTGATCG |
|  | Reverse | CCACTTGGTGGTTTGTGAGTG |
| rats *MCP-1* | Forward | CCAATGAGTAGGCTGGAGAGC |
|  | Reverse | ACCCATTCCTTCTTGGGGTC |

**Table S1. Sequences of primers used for real-time RT-PCR.** IL-6, interleukin 6; IL-17, interleukin 17; TNF-α, tumor necrosis factor-α; MCP-1, monocyte chemoattractant protein-1.
